# Is electronic cigarette use a risk factor for stroke? A systematic review and meta-analysis

**DOI:** 10.18332/tid/154364

**Published:** 2022-11-14

**Authors:** Kai Zhao, Jing Li, Ping Zhou, Ling Xu, Mingfei Yang

**Affiliations:** 1 Graduate School, Qinghai University, Xining, People's Republic of China; 2 Department of Community Health Education, Institute for Health Education of Qinghai Province, Xining, People's Republic of China; 3 Department of Neurosurgery, Qinghai Provincial People's Hospital, Xining, People's Republic of China

**Keywords:** electronic cigarette, stroke, meta-analysis, odds ratio

## Abstract

**INTRODUCTION:**

Stroke, as a common cerebrovascular disease, has a high mortality and disability rate. Although many studies have reported that using e-cigarettes was associated with occurrence of stroke, some studies have concluded that e-cigarettes may help smokers stop using combustible cigarettes and reduce the risk of stroke. Therefore, we aimed to validate the hypothesis that e-cigarette use might be an independent risk factor for stroke occurrence by performing a systematic review and meta-analysis of clinical epidemiology studies.

**METHODS:**

The pooled effect was calculated by the random effects model. I^2^ was used to test for heterogeneity. Sensitivity analysis was performed to evaluate the stability of the overall results. Funnel plot symmetry or Egger’s regression was used to evaluate publication bias. All p values were two-sided with significance level at 0.05.

**RESULTS:**

Six cross-sectional studies with high quality were finally included in the meta-analysis, which included a total of 1134896 participants. Analysis with random effects model showed that the total pooled odds ratio (OR) of stroke occurrence in e-cigarette users was 1.25 (95% CI: 1.01–1.55) (I^2^=96.6%, p<0.001). A stable result was revealed by sensitivity analysis. There was no publication bias. Due to high heterogeneity, we performed subgroup analysis. Compared to neither e-cigarette nor combustible cigarette users, pooled OR of stroke occurrence in e-cigarette only users was 1.13 (95% CI: 0.99–1.29) (I^2^=45.9%, p=0.116). Compared to combustible cigarette only users, pooled OR of stroke occurrence in both of e-cigarette and combustible cigarette users was 1.39 (95% CI: 1.19–1.64) (I^2^=5.6%, p=0.303). In addition, pooled OR in currently e-cigarette only users who were formerly combustible cigarette only users was 1.59 (95% CI: 1.22–2.07) (I^2^=0.0%, p=0.989).

**CONCLUSIONS:**

The role of e-cigarette use in the development of stroke is inconclusive, due to the strong effect of prior tobacco use as a risk factor for stroke in the included studies.

## INTRODUCTION

Electronic cigarettes (e-cigarettes) are an electronic product consisting of 3 major components: a rechargeable battery, a smoke producing device, and an inhalation box^1^. The ingredients of inhalation mainly consist of edible glycerol, edible propanediol, edible propanetriol and edible essential oils^2^. Compared to combustible cigarettes, e-cigarettes have no tar, nicotine, or carbon monoxide in inhalation. Therefore, the initial purpose of e-cigarettes is to help smokers or nicotine dependents quit smoking and reduce organ damage caused by smoking^3^. To obtain a more realistic feeling as if smoking combustible cigarettes, different proportions of nicotine are added to e-cigarettes^4^. In addition, a plethora of design features have led to an increase in smoking e-cigarettes among youth^5^. However, e-cigarette use may be related to other substances^6,7^. Ingredients of smoke produced by e-cigarettes may not only injure the lung but also can enter the circulatory system via gas exchange in alveoli, thus inducing cardiovascular risk^8,9^.

Stroke, as a common cerebrovascular disease, has a high mortality and disability rate. Although many studies have reported that using e-cigarettes was associated with occurrence of stroke, some studies have concluded that e-cigarettes may help smokers stop using combustible cigarettes and reduce the risk of stroke^10,11^. Therefore, we aimed to perform a systematic review and meta-analysis of clinical epidemiology studies that assessed the association between stroke and e-cigarette use.

## METHODS

This systematic review and meta-analysis was performed referring to the protocol published in the database of International Platform of Registered systematic review and Meta-analysis Protocols (INPLASY, https://inplasy.com/, registration number: INPLASY202180086, DOI number: 10.37766/inplasy2021.8.0086.)

### Search strategy

Literature search was performed in three public electronic databases of PubMed, Embase and Cochrane. The strategy of literature search is available in the Supplementary file.

### Data extraction

Before data extraction, the quality assessment of included articles was performed via the Newcastle-Ottawa Quality Assessment Scale Cohort Studies (NOQAS-CO) for cohort studies, Newcastle-Ottawa Quality Assessment Scale Case-control Studies (NOQAS-CA) for case-control studies, and Agency for Healthcare Research and Quality (AHRQ) for cross-sectional studies. For studies with the same quality assessed by the above evaluation standards, studies with larger number of included patients were considered to have higher quality. Under the premise that e-cigarette use was an independent exposure factor, all the data used to assess risk degree of stroke occurrence were extracted, including hazard ratio (HR), risk ratio (RR) in a prospective observational study, and odds ratio (OR) in a retrospective observational study. In addition, some confounders, which might result in errors, were adjusted, including gender, age, solutions of combustible cigarette smoking and e-cigarette use, definition of endpoints, period of observation, and other factors.

### Study selection

Inclusion criteria were: 1) language, region or publication year, were not restricted; 2) clinical epidemiological studies included a cross-sectional study, a case-control study, and cohort study; 3) exposed group and non-exposed group differed in e-cigarette use; 4) baseline characteristics were not statistically different between exposed group and non-exposed group; 5) endpoint of observation was stroke; 6) a complete analysis of the outcomes of cohort studies was performed. Exclusion criteria were: 1) duplication; 2) reviews, comments, letters, case reports, protocols, notes and conference papers; 3) animal experiments; and 4) contents of articles that were irrelevant to this meta-analysis.

### Meta-analysis

Relative numbers and their 95% confidence intervals were used to describe count data. Meta-analysis was performed using corresponding modules in Software for Statistics and Data Science (Stata, version 15.1; College Station, Texas 77845 USA). The pooled effect with 95% CI was calculated by a random effects model. I^2^ was used to test for heterogeneity. Sensitivity analysis was performed to evaluate the stability of overall results by recalculating the pooled effect of the remaining studies after omitting the study with the highest quality or the random effects model was switched to fixed effects model. Funnel plot symmetry or Egger’s regression was used to evaluate publication bias. To reduce heterogeneity, we recalculated the pooled effect of the remaining studies after omitting the study with the lowest quality or perform subgroup analysis directly. All p values were two-sided with a significance level set at 0.05.

### Patient and public involvement

There were no patients or applicable public involved in this review.

## RESULTS

**Figure 1:**
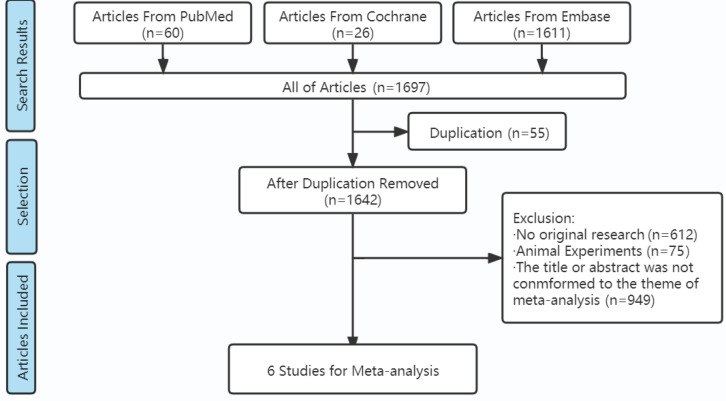
Process of literature search

Totally, 1697 articles were retrieved from 3 databases according to the search strategy. After further screening according to the inclusion and exclusion criteria, 6 articles^11-16^ of cross-sectional studies were finally included (Figure 1). There were 1134896 participants in the 6 included studies (Table 1). The age range was 15–78 years. Males were close to 50%. Periods of observation were from 1 to 4 years. Different solutions of combustible cigarette smoking and e-cigarette use could be classified into 6 types: 1) neither e-cigarette nor combustible cigarette use; 2) currently e-cigarette use only but formerly combustible cigarette use only; 3) combustible cigarette use only; 4) e-cigarette use only; 5) both of e-cigarette and combustible cigarette use; and 6) solutions of combustible cigarette smoking and e-cigarette use were not separated, including 2), 4) and 5). Thus, different data were extracted from the same article. One study (2021 Reynolds) divided the type 6) solution into 3 sub-solutions: 1. Frequency of e-cigarette use > frequency of combustible cigarette smoking, 2. Frequency of e-cigarette use < frequency of combustible cigarette smoking, and 3. Frequency of e-cigarette use = frequency of combustible cigarette smoking, which produced 3 data extractions. One study (2021 Bricknell) divided the type 4) solution into 3 sub-solutions: e-cigarette use only every day, e-cigarette use only sometimes, e-cigarette use formerly.

**Table 1:** Characters of studies included

*Articles*	*Male %*	*Age (years)*	*Participants n*	*Solutions of combustible cigarette smoking and e-cigarette use*	*Time of observation*	*Endpoints of observation*
2019 Osei	48.10	30–34	449092	1. Combustible cigarette use only vs both of e-cigarette and combustible cigarette use	2	Stroke
				2. Neither e-cigarette nor combustible cigarette use vs both of e-cigarette and combustible cigarette use		
				3. Currently e-cigarette use only but formerly combustible cigarette use only vs combustible cigarette use only		
2019 Parekh	47.90	18-44	161529	1. Neither e-cigarette nor combustible cigarette use vs e-cigarette use only	2	Stroke
				2. Neither e-cigarette nor combustible cigarette use vs currently e-cigarette use only but formerly combustible cigarette use only		
				3. Neither e-cigarette nor combustible cigarette use vs both of e-cigarette and combustible cigarette use		
				4. Combustible cigarette use only vs e-cigarette use only		
				5. Currently e-cigarette use only but formerly combustible cigarette use only vs combustible cigarette use only		
				6. Combustible cigarette use only vs both of e-cigarette and combustible cigarette use		
2020 Sverre	80.60	64-78	1789	1. Neither e-cigarette nor combustible cigarette use vs solutions of combustible cigarette smoking and e-cigarette use were not separated	2	Stroke or Transitory ischemic attacks
2021 Reynolds	51.50	≥18	32172	1. Neither e-cigarette nor combustible cigarette use vs frequency of e-cigarette use < frequency of combustible cigarette smoking	4	Stroke
				2. Neither e-cigarette nor combustible cigarette use vs frequency of e-cigarette use = frequency of combustible cigarette smoking		
				3. Neither e-cigarette nor combustible cigarette use vs frequency of e-cigarette use < frequency of combustible cigarette smoking		
				4. Neither e-cigarette nor combustible cigarette use vs e-cigarette use only		
				5. Neither e-cigarette nor combustible cigarette use vs both of e-cigarette and combustible cigarette use		
2021 Jankowski	47.90	≥15	1011	1. Neither e-cigarette nor combustible cigarette use vs solutions of combustible cigarette smoking and e-cigarette use were not separated	1	Stroke
2021 Bricknell	43.20	≥18	489303	1. Neither e-cigarette nor combustible cigarette use vs e-cigarette use only every day	1	Stroke
				2. Neither e-cigarette nor combustible cigarette use vs e-cigarette use only sometimes		
				3. Neither e-cigarette nor combustible cigarette use vs e-cigarette use only formerly		

Because all the articles were cross-sectional studies, only AHRQ was used to assess their quality. One study (2019 Osei) was assessed as ‘Yes’ in every section, which had the highest quality (Table 2). In subjective influence section, one article (2020 Sverre) was assessed as ‘No’. Five articles were assessed as ‘No’ in the quality assurance section. In the data missed section, two articles were assessed as ‘No’. All the studies were assessed as ‘No’ in the follow-up section. Finally, one article (2020 Sverre) was assessed to have 6 ‘Yes’, which had the lowest quality. Only ORs directly appeared in articles, which were extracted by us to perform the next stage of data analysis.

**Table 2:** Quality assessment of studies via Agency for Healthcare Research and Quality

*Articles*	*Source of data*	*Inclusion and Exclusion criteria*	*Identification time*	*Continuous participants*	*Subjective effects*	*Quality assurance*	*Causes of exclusion*	*Confounders*	*Data missed*	*Data integrality*	*Follow-up*
2019 Osei	Yes	Yes	Yes	Yes	Yes	Yes	Yes	Yes	Yes	Yes	**No**
2019 Parekh	Yes	Yes	Yes	Yes	Yes	**No**	Yes	Yes	Yes	Yes	**No**
2020 Sverre	Yes	Yes	Yes	Yes	**No**	**No**	Yes	Yes	**No**	Yes	**No**
2021 Reynolds	Yes	Yes	Yes	Yes	Yes	**No**	Yes	Yes	Yes	Yes	**No**
2021 Jankowski	Yes	Yes	Yes	Yes	Yes	**No**	Yes	Yes	**No**	Yes	**No**
2021 Bricknell	Yes	Yes	Yes	Yes	Yes	**No**	Yes	Yes	Yes	Yes	**No**

**Figure 2:**
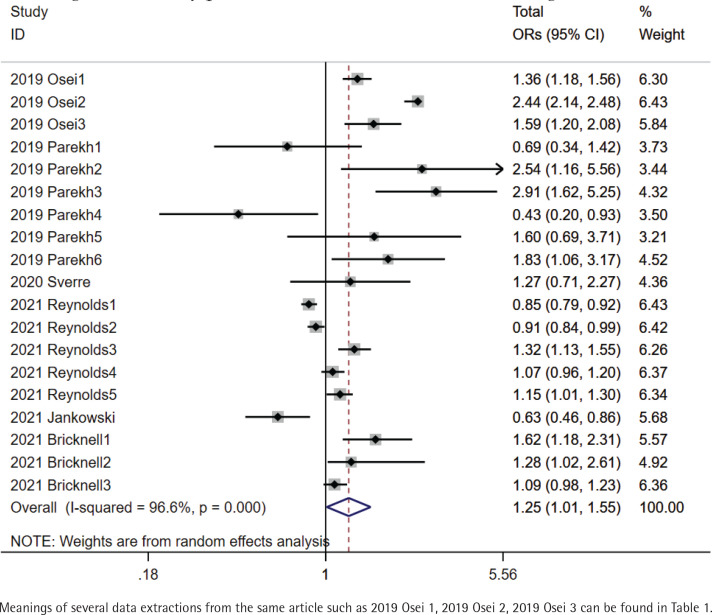
Totally pooled OR of stroke occurrence in e-cigarette users

**Figure 3:**
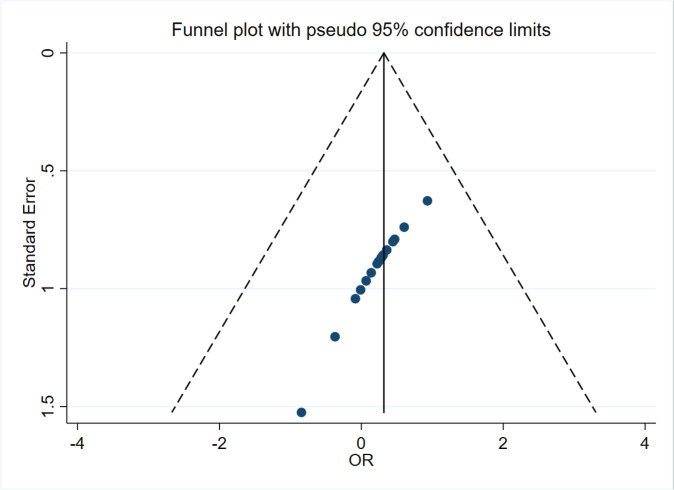
Funnel plot of all the ORs extracted from articles

**Figure 4:**
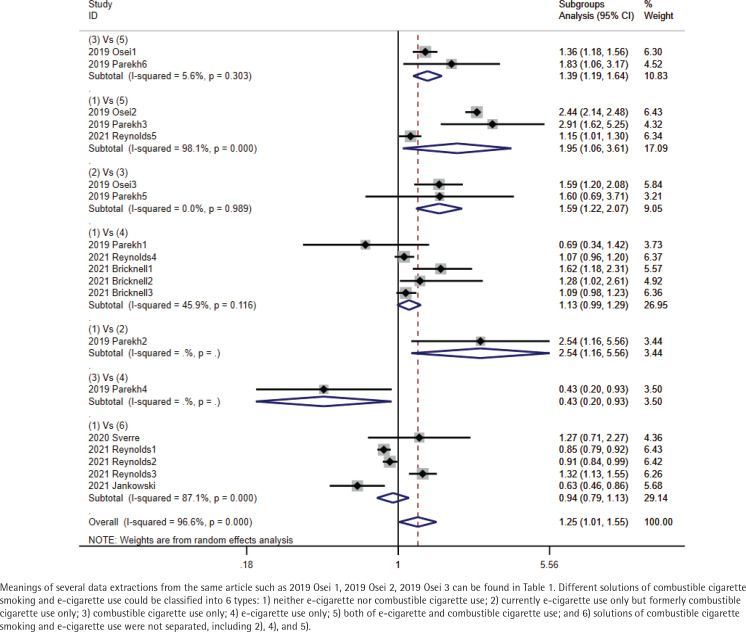
Subgroups analysis of totally pooled OR

The total pooled OR was 1.25 (95% CI: 1.01–1.55) with heterogeneity of I^2^=96.6% (p<0.001) (Figure 2). In the sensitivity analysis, after omitting the study with the highest quality (2019 Osei), pooled OR was 0.99 (95% CI: 0.96–1.04) with a heterogeneity of I^2^=83.4% (p<0.001). There was a symmetrical distribution in the funnel plot (Figure 3). The study with the lowest quality (2020 Sverre) might have resulted in the high heterogeneity of total pooled OR; after omitting this study, the pooled OR was 1.23 (95% CI: 1.19–1.28) with a heterogeneity of I^2^=96.8% (p<0.001). However, heterogeneity was still high. Referring to combined combustible cigarette smoking and e-cigarette use, we performed subgroup analysis of total ORs. Compared to neither e-cigarette nor combustible cigarette users, pooled OR of stroke occurrence in current e-cigarette only users was 1.13 (95% CI: 0.99–1.29) (I^2^=45.9%, p=0.116). Compared to combustible cigarette only smokers, pooled OR of stroke occurrence in dual e-cigarette and combustible cigarette users was 1.39 (95% CI: 1.19–1.64) (I^2^=5.6%, p=0.303). In addition, pooled OR in currently e-cigarette only users who were formerly combustible cigarette only smokers was 1.59 (95% CI: 1.22–2.07) (I^2^=0.0%, p=0.989). Compared to neither e-cigarette nor combustible cigarette users, pooled OR of stroke occurrence in e-cigarette users who did not clearly distinguish the use of combustible cigarettes was 0.94 (95% CI: 0.79–1.13) and pooled OR in both e-cigarette and combustible cigarette users was 1.95 (95% CI: 1.06–3.61) with a heterogeneity of I^2^=87.1% (p<0.001) and I^2^=98.1% (p<0.001) (Figure 4). Compared to combustible cigarette only smokers, only one OR of stroke occurrence in e-cigarette only users was 0.43 (95% CI: 0.20–0.93). Compared to neither e-cigarette nor combustible cigarette users, pooled OR in current e-cigarette only users who were formerly combustible cigarette only smokers was 2.54 (95% CI: 1.16–5.56).

## DISCUSSION

Our results of total pooled OR showed that the role of e-cigarettes as a risk factor for stroke is inconclusive due to the strong effect of prior tobacco use. However, publication bias and the data from the study with the lowest quality were not the source of significant heterogeneity. Finally, results in subgroup analysis support that one source of heterogeneity was the ambiguous solutions of combustible cigarette smoking and e-cigarette use. Moreover, due to the specific limitations of the cross-sectional study, we could not judge the causal and chronological ordering relationship of e-cigarette use and other risk factors of stroke occurrence such as hypertension, diabetes, auricular fibrillation, which might be other sources of heterogeneity. Compared to combustible cigarette only use, current e-cigarette only users with formerly combustible cigarette only smoking would face the risk of stroke occurrence, which might confirm that e-cigarette use as a replacement or adjunctive therapy for quitting smoking, could not reduce the risk of stroke occurrence. However, compared to neither e-cigarette nor combustible cigarette users, e-cigarette only use might not be the risk factor for stroke occurrence.

Smoke produced by e-cigarettes could injure the blood-brain barrier^17,18^ and lead to neuroinflammation^19^. In addition, e-cigarette exposure could influence cognitive functions^20^ and decrease brain glucose utilization in ischemic stroke^20^, which might lead to an unfavorable prognosis for stroke patients. We considered that blood vessel endothelia had been damaged via smoking combustible cigarettes. E-cigarette use might further deteriorate the injury of cerebrovascular endothelium in current e-cigarette users who smoked combustible cigarettes currently or formerly. Although e-cigarette only use might not be the risk factor for stroke, it might be associated with other diseases such as cancer, heart and lung diseases^21,22^. Therefore, the better choice for quitting smoking or nicotine might be to immediately stop using e-cigarettes and smoking combustible cigarettes, which might be a better way to reduce organ injuries.

### Limitations

The major limitation of our study was that although there were sufficient data from 6 studies, the quality of the data could have been higher. Second, stroke as the definition of endpoint covers a wide range. Subtypes including transient ischemic attack, hemorrhagic stroke, and ischemic stroke could not be clearly separated. In addition, only ORs were extracted, which were of lower quality to explain causal relationships. Cohort studies that include e-cigarette users with no history of tobacco use are needed to confirm if e-cigarettes are an independent factor for stroke, an assessment which cannot be currently made.

## CONCLUSIONS

The role of e-cigarette use on the development of stroke is inconclusive, due to the strong effect of prior tobacco use as a risk factor for stroke, in the included studies.

## Data Availability

The data supporting this research are available from the authors on reasonable request.
