# Supplementary material for: Is electronic cigarette use a risk factor for stroke? A systematic review and meta-analysis
Source: Tob Induc Dis. 2022 Nov 14;20:101. doi: 10.18332/tid/154364 (PMC9661377; doi:10.18332/tid/154364)
Supplement: Supplementary file 1 [file TID-20-101-s1.pdf]

Supplementary file

(((((((((Electronic Nicotine Delivery System[Title/Abstract]) OR (E-Cigs*[Title/Abstract])) OR (E Cig*[Title/Abstract])) OR (E-Cigarette*[Title/Abstract])) OR (E Cigarette*[Title/Abstract])) OR (Electronic Cigarette*[Title/Abstract])) OR (Cigarette*, Electronic[Title/Abstract])) OR (Electronic Nicotine Delivery Systems/exp))) AND (((((((((((((((((((((((((Stroke/exp) OR (Stroke*[Title/Abstract])) OR (Cerebrovascular Accident*[Title/Abstract])) OR (CVA* (Cerebrovascular Accident)[Title/Abstract])) OR (Cerebrovascular Apoplexy[Title/Abstract])) OR (Apoplexy, Cerebrovascular[Title/Abstract])) OR (Vascular Accident, Brain[Title/Abstract])) OR (Brain Vascular Accident*[Title/Abstract])) OR (Vascular Accidents, Brain[Title/Abstract])) OR (Cerebrovascular Stroke*[Title/Abstract])) OR (Stroke*, Cerebrovascular[Title/Abstract])) OR (Apoplexy[Title/Abstract])) OR (Cerebral Stroke*[Title/Abstract])) OR (Stroke*, Cerebral[Title/Abstract])) OR (Stroke, Acute[Title/Abstract])) OR (Acute Stroke*[Title/Abstract])) OR (Strokes, Acute[Title/Abstract])) OR (Cerebrovascular Accident*, Acute[Title/Abstract])) OR (Acute Cerebrovascular Accident*[Title/Abstract])))) OR ((((((((((((((((((((((((((((Ischemic Attack, Transient/exp) OR (TIA* (Transient Ischemic Attack[Title/Abstract]))) OR (Transient Ischemic Attack*[Title/Abstract])) OR (Attack*, Transient Ischemic[Title/Abstract])) OR (Ischemic Attacks, Transient[Title/Abstract])) OR (Brain TIA[Title/Abstract])) OR (TIA, Brain[Title/Abstract])) OR (Carotid Circulation Transient Ischemic Attack[Title/Abstract])) OR (Transient Ischemic Attack, Carotid Circulation[Title/Abstract])) OR (Transient Ischemic Attack, Vertebrobasilar Circulation[Title/Abstract])) OR (Vertebrobasilar Circulation Transient Ischemic Attack[Title/Abstract])) OR (Crescendo Transient Ischemic Attacks[Title/Abstract])) OR (Transient Ischemic Attacks, Crescendo[Title/Abstract])) OR (Posterior Circulation Transient Ischemic Attack[Title/Abstract])) OR (Transient Ischemic Attack, Posterior Circulation[Title/Abstract])) OR (Transient Ischemic Attack, Anterior Circulation[Title/Abstract])) OR (Anterior Circulation Transient Ischemic Attack[Title/Abstract])) OR (Brain Stem Ischemia, Transient[Title/Abstract])) OR (Transient Ischemic Attack, Brain Stem[Title/Abstract])) OR (Brainstem Ischemia*, Transient[Title/Abstract])) OR (Ischemia*, Transient Brainstem[Title/Abstract])) OR (Transient Brainstem Ischemia[Title/Abstract])) OR (Brainstem Transient Ischemic Attack[Title/Abstract])) OR (Transient Ischemic Attack, Brainstem[Title/Abstract])) OR (Brain Stem Transient Ischemic Attack[Title/Abstract])) OR (Cerebral Ischemia*, Transient[Title/Abstract])) OR (Ischemia*, Transient Cerebral[Title/Abstract])) OR (Transient Cerebral Ischemia*[Title/Abstract]))) OR (((((((((((((Cerebral Hemorrhage/exp) OR (Hemorrhage*, Cerebrum[Title/Abstract])) OR (Cerebrum Hemorrhage*[Title/Abstract])) OR (Cerebral Parenchymal Hemorrhage*[Title/Abstract])) OR (Hemorrhage*, Cerebral Parenchymal[Title/Abstract])) OR (Parenchymal Hemorrhage*, Cerebral[Title/Abstract])) OR (Intracerebral Hemorrhage*[Title/Abstract])) OR

(Hemorrhage*, Intracerebral[Title/Abstract])) OR (Hemorrhage*, Cerebral[Title/Abstract])) OR (Cerebral Hemorrhages[Title/Abstract])) OR (Brain Hemorrhage*, Cerebral[Title/Abstract])) OR (Cerebral Brain Hemorrhage*[Title/Abstract])) OR (Hemorrhage*, Cerebral Brain[Title/Abstract]))) OR (((((((((((((((Subarachnoid Hemorrhage/exp) OR (SAH* (Subarachnoid Hemorrhage[Title/Abstract]))) OR (Hemorrhage*, Subarachnoid[Title/Abstract])) OR (Subarachnoid Hemorrhages[Title/Abstract])) OR (Subarachnoid Hemorrhage*, Aneurysmal[Title/Abstract])) OR (Aneurysmal Subarachnoid Hemorrhage*[Title/Abstract])) OR (Hemorrhage*, Aneurysmal Subarachnoid[Title/Abstract])) OR (Subarachnoid Hemorrhage*, Spontaneous[Title/Abstract])) OR (Hemorrhage*, Spontaneous Subarachnoid[Title/Abstract])) OR (Spontaneous Subarachnoid Hemorrhage*[Title/Abstract])) OR (Perinatal Subarachnoid Hemorrhage*[Title/Abstract])) OR (Hemorrhage*, Perinatal Subarachnoid[Title/Abstract])) OR (Subarachnoid Hemorrhage*, Perinatal[Title/Abstract])) OR (Subarachnoid Hemorrhage*, Intracranial[Title/Abstract])) OR (Hemorrhage*, Intracranial Subarachnoid[Title/Abstract])) OR (Intracranial Subarachnoid Hemorrhage*[Title/Abstract]))) OR ((((((((((((((Cerebrovascular Disorders/exp) OR (Cerebrovascular Disorder[Title/Abstract])) OR (Vascular Disease*, Intracranial[Title/Abstract])) OR (Intracranial Vascular Disease*[Title/Abstract])) OR (Intracranial Vascular Disorder*[Title/Abstract])) OR (Vascular Disorder*, Intracranial[Title/Abstract])) OR (Cerebrovascular Disease*[Title/Abstract])) OR (Disease*, Cerebrovascular[Title/Abstract])) OR (Brain Vascular Disorder*[Title/Abstract])) OR (Vascular Disorder*, Brain[Title/Abstract])) OR (Cerebrovascular Occlusion*[Title/Abstract])) OR (Occlusion*, Cerebrovascular[Title/Abstract])) OR (Cerebrovascular Insufficiency[Title/Abstract])) OR (Cerebrovascular Insufficiencies[Title/Abstract])) OR (Insufficiencies, Cerebrovascular[Title/Abstract])) OR (Insufficiency, Cerebrovascular[Title/Abstract]))).

© 2022 Zhao K. et al.
